# *PIEZO1* Is Selectively Expressed in Small Diameter Mouse DRG Neurons Distinct From Neurons Strongly Expressing *TRPV1*

**DOI:** 10.3389/fnmol.2019.00178

**Published:** 2019-07-19

**Authors:** Jigong Wang, Jun-Ho La, Owen P. Hamill

**Affiliations:** Department of Neuroscience, Cell Biology and Anatomy, The University of Texas Medical Branch, Galveston, TX, United States

**Keywords:** *PIEZO1*, *PIEZO2*, *TRPV1*, Yoda1, mechanically gated channel, mechano-nociception, pain

## Abstract

Using a high resolution *in situ* hybridization technique we have measured *PIEZO1*, *PIEZO2*, and *TRPV1* transcripts in mouse dorsal root ganglion (DRG) neurons. Consistent with previous studies, *PIEZO2* transcripts were highly expressed in DRG neurons of all sizes, including most notably the largest diameter neurons implicated in mediating touch and proprioception. In contrast, *PIEZO1* transcripts were selectively expressed in smaller DRG neurons, which are implicated in mediating nociception. Moreover, the small neurons expressing *PIEZO1* were mostly distinct from those neurons that strongly expressed *TRPV1*, one of the channels implicated in heat-nociception. Interestingly, while *PIEZO1-* and *TRPV1-* expressing neurons form essentially non-overlapping populations, *PIEZO2* showed co-expression in both populations. Using an *in vivo* functional test for the selective expression, we found that Yoda1, a PIEZO1-specific agonist, induced a mechanical hyperalgesia that displayed a significantly prolonged time course compared with that induced by capsaicin, a TRPV1-specific agonist. Taken together, our results indicate that PIEZO1 should be considered a potential candidate in forming the long sought channel mediating mechano-nociception.

## Introduction

Mechanical forces can evoke many different types of sensation beginning in the peripheral nervous system, including discriminative touch, proprioception, and mechano-nociception. Recent studies have provided strong evidence that the mechanically gated channel PIEZO2 mediates discriminative touch and proprioception, but not mechano-nociception (Coste et al., 2010; Ranade et al., 2014; Woo et al., 2014, 2015; Florez-Paz et al., 2016). In particular, transgenic mice lacking PIEZO2 show a profound loss of touch and proprioception, but show little or no impairment in their normal ability to detect painful mechanical stimuli (Ranade et al., 2014; Woo et al., 2015; Murthy et al., 2018). The mouse results have recently been confirmed in human patients that display a genetic loss of PIEZO2. In particular, these patients show a general loss of vibration detection, touch discrimination and joint proprioception, while retaining almost normal thresholds for mechanical pain (Chesler et al., 2016; Mahmud et al., 2016; Vedove et al., 2016; Szczot et al., 2018). Therefore, one of most critical sensory mechanisms involved in determining how an animal senses and responds to its surroundings, namely the mechano-nociceptive channel, remains to be identified (Hu et al., 2006; Woolf and Ma, 2007; Dubin and Patapoutian, 2010; Murthy et al., 2018; Szczot et al., 2018). One possible candidate may be the closely related mechanically gated channel PIEZO1 (Coste et al., 2010). However, initial *PIEZO* expression studies using RT-PCR indicated that while *PIEZO2* was highly expressed in mouse DRG neurons, *PIEZO1* transcripts were barely detectable (Coste et al., 2010). Moreover, this apparent *PIEZO1* absence was confirmed by *in situ* hybridization (ISH) measurements (Ranade et al., 2014). However, another group studying the mechanosensory facial organ of the star-nosed mole, found using qPCR that *PIEZO1* was detected at significant levels in both the mole’s trigeminal ganglia (TG) and DRG. Moreover, *PIEZO1* was enriched over *PIEZO2* in neurons, not only in the star-nosed mole TG, but also in the mouse TG (Gerhold et al., 2013). Interestingly, other evidence suggesting a PIEZO role in mechano-nociception has come from studies of *Drosophila* where knockout of the single *PIEZO* homolog blocks mechano-nociception (Kim et al., 2012). What remains unclear is whether this ancestral function has been conserved in vertebrates. Because the issue of *PIEZO1* expression and its somatosensory function in vertebrates remains unclear, we decided to reinvestigate *PIEZO1* expression in mouse DRG, taking special notice of its possible expression in small diameter DRG neurons that are generally implicated as mediating nociception (Lee et al., 1986; Lawson and Waddell, 1991; Le Pichon and Chesler, 2014). In brief, our results indicate that *PIEZO1* is selectively expressed in small diameter neurons and these neurons are mostly distinct from those neurons strongly expressing *TRPV1*, a channel implicated in mediating heat nociception (Caterina et al., 1997). Moreover, we found that Yoda1, a highly specific agonist for PIEZO1 over PIEZO2 (Syeda et al., 2015), induced a nociceptive response (hyperalgesia) in mice that was significantly prolonged in time course compared with the hyperalgesia induced by capsaicin, a TRPV1 agonist (Caterina et al., 1997). Taken together these results implicate PIEZO1 in forming the long sought mechano-nociceptor channel. In direct support of this idea and while this manuscript was under review, a Finnish group (Mikhailov et al., 2019) reported that PIEZO1 proteins are expressed in mouse trigeminal cultured neurons and that Yoda1 induces rapid Ca^2+^ transients in isolated trigeminal neurons. Even more compelling, Yoda1 was shown to induce, in a rat hemi-skull preparation, a pronounced and sustained firing of trigeminal mechanosensory nerve fibers innervating the meninges. Based on these results it was concluded that PIEZO1 plays a crucial role in triggering pulsating migraine related nociception (Mikhailov et al., 2019).

## Materials and Methods

### Preparation of Mouse Dorsal Root Ganglia

All experimental protocols were approved by the Animal Care and Use Committee at the UTMB and are in accordance with the NIH *Guide for the Care and Use of Laboratory Animals*. Young adult FVB/NJ male mice 4–5-week old, 20–25 g body weight, (The Jackson Laboratory, Bar Harbor, ME, United States) were used for both in ISH and behavioral studies (see also Schwartz et al., 2008). For the ISH studies, the mice were deeply anesthetized with isoflurane and perfused through the aorta, firstly with cold heparinized and then 10% formalin in phosphate-buffered saline (PBS). Their DRG were harvested from all spinal levels and fixed in 10% formalin overnight. The DRG were then dehydrated through an ethanol series/xylene and embedded in paraffin.

### *In situ* Hybridization

Ten micrometer sections of DRG were cut and *in situ* hybridization was carried out using the RNAscope assay according to the manufacturer’s instructions (Advanced Cell Diagnostics, Hayward, CA, United States). The RNAscope technique is able to assess cellular RNA content with single molecule resolution within individual cells through the use of a novel probe design strategy and a hybridization-based signal amplification system that simultaneously amplifies signals and suppresses background (Wang et al., 2012). Development of signal was done using the RNAscope 2.0 HD brown detection kit. Probes for *mPIEZO1* (cat number: 400181) and *mPIEZO2* (cat number: 400191) and *mTRPV1* (cat number: 313331) were purchased from ACD (Hayward, CA, United States). The *PIEZO1* probe involved NT 5477-6623 (corresponding to amino acids 1825-2207). The *PIEZO2 in situ* probe involved NT 983-1920 (amino acids 328-64). As a positive control for RNA integrity, an RNAscope probe specific to the house keeping gene for peptidyl-prolyl isomerase B (*PPIB*) RNA (cat number: 313911) was used. *PPIB* has been recommended by RNAscope because it is expressed at a sufficiently low level in all cell types so as to provide a rigorous control for sample quality and technical performance. As a negative control, a probe specific to bacterial dihydrodipicolinate reductase (*dapB)* RNA (cat number: 310043) was used. The negative control ensured that there was no background staining related to the assay and DRG specimen. The ISH results are based on detailed analysis of 891 neurons from ∼50 DRG isolated from three different mice.

Slides were mounted with Cytoseal and imaged under a bright field Olympus BX51 microscope (10×, 40×, and 60× objectives) with Olympus DP imaging software. The cross sectional area of neurons was measured using the Image J software^1^. Bright puncta (or dots) rather than a diffuse staining pattern represent true RNA transcript signals and *PIEZO1* transcripts were counted within each defined cell area. Both *PIEZO1* and *PPIB* staining most typically appeared as single puncta, or less frequently as clusters of 2–3 puncta distributed throughout the neurons, enabling the direct count of transcripts. *PIEZO2* and *TRPV1* staining more often appeared in clumps, presumably representing many superimposed stained transcripts. In these cases in order to obtain an estimate of the transcript density within the clumps, Image J was used to measure the clump area, which was then divided by the area of individual punctate stains in relatively low transcript density regions. This method may underestimate density because of overlying transcripts. In order to estimate expression of *PIEZO1*, *PIEZO1*, and/or *TRPV1* within the same DRG neuron, sequential DRG slices were stained by different probes (i.e., there was no re-staining of a slice with multiple probes) and the same neuron was identified by its similar size, shape and neighbors, allowing for some cell distortion and reorientation caused by the slicing.

### Testing the Behavioral Response to Yoda1 and Capsaicin Injections

Mice were housed in groups of four to five in plastic cages with soft bedding and free access to food and water under a 12-12-h light–dark cycle. All animals were acclimated for 1 week before any experimental procedures. To compare Yoda1 (Tocris, Minneapolis, MN, United States) with well-established nociceptive responses caused by capsaicin injections (Schwartz et al., 2008, 2009), each chemical was injected into the footpads of different mice. One micromole of Yoda1 was dissolved in 1 ml dimethyl sulfoxide and three micromole capsaicin in 1 ml of vehicle containing 20% alcohol and 10% Tween 80 in saline, immediately before injection.

For behavioral experiments each mouse was anesthetized with isoflurane (4% for induction and 1.5% for maintenance) in a flow of O_2_ and placed in a prone position, and then 5 μl of either Yoda1 (i.e., 5 nanomole) or capsaicin (i.e., 15 nanomole) solutions was injected intra-dermally using a 30 gauge needle attached to a Hamilton syringe (i.e., to give maximum local dermal concentrations of 1 mM Yoda1 and 3 mM capsaicin). As a control for the vehicle and the injection, the same volume of vehicle alone (i.e., used for Yoda1 or capsaicin) was injected in different mice. In each case, the needle was inserted near the heel of the left hind foot and advanced to the middle of the plantar surface (Figure 9A). The insertion site was pressed for 1 min to prevent leakage of the solution after removal of the needle. Anesthesia was discontinued and the mice were aroused within 5 min and then returned to their cages. For behavioral testing the mice were placed on an elevated metal grid and mechanically stimulated by applying punctate stimulation on the hind paw plantar with a von Frey filament (VFF) which was equivalent to 0.1 g force. Foot withdrawal frequencies in response to the VFF stimuli were measured as an indicator of mechanical hyperalgesia. To assess primary hyperalgesia the VFF was applied to a site <3 mm distal from the injection site. For secondary hyperalgesia, the VFF was applied at the base and/or proximal part of the third and fourth toes (see Figure 9A, Schwartz et al., 2008). This area is considered an adequate distance from the injection site, and thus should not be directly affected by the injection. Effects of Yoda1 or capsaicin on foot withdrawal responses were assessed before and 0.5, 1, 2, 3, 4, 6, 24, and 48 h after intradermal injection for both chemicals (tested blindly against the vehicle alone) and for Yoda1 also after 72 and 96 h that was necessary to observe full recovery. The Mann–Whitney *U* test was used to compare Yoda1 or capsaicin against vehicle at each time point.

## Results

### *PIEZO1* and *PIEZO2* Expression Patterns in Mouse DRG

**FIGURE 1:**
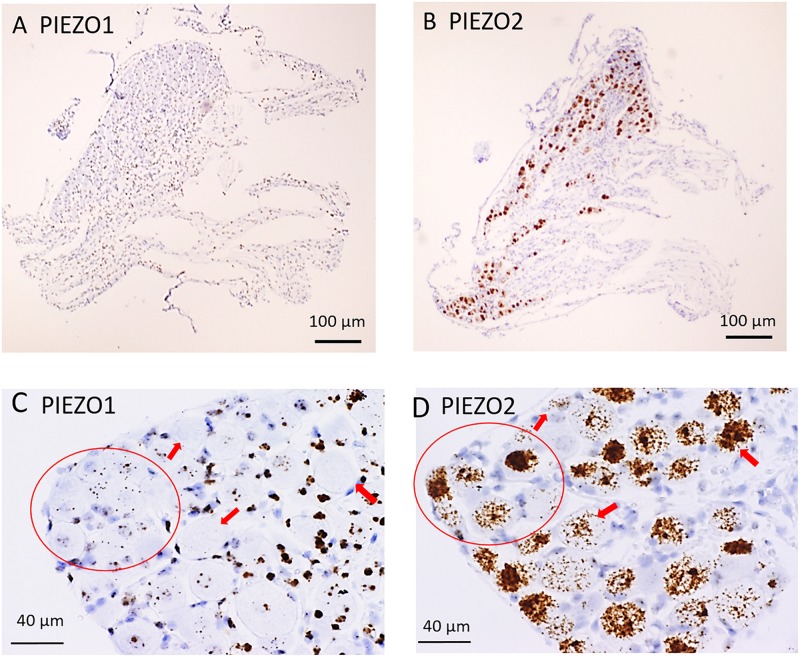
*PIEZO1* and *PIEZO2* expression in mouse DRG. **(A,B)** Adjacent DRG slices viewed at low magnification (10× objective) showing faint brown punctuate staining by the *PIEZO1* RNA probe **(A)** and dark brown staining with the *PIEZO2* probe **(B)**. **(C,D)** Specific DRG regions examined at higher magnification (40× objective). The red circles circumscribe several (∼10) small DRG neurons with punctuate *PIEZO1* probe staining **(C)** as well as five larger neurons with denser *PIEZO2* probe staining **(D)**. The red arrows indicate large neurons with variable densities of *PIEZO2* probe staining **(D)** but no staining by the *PIEZO1* probe **(C)**.

Figure 1 shows microscopic images of slices from the same DRG examined at low (Figures 1A,B) and higher magnification (Figures 1C,D) stained with RNA probes for *PIEZO1* and *PIEZO2*. Whereas the *PIEZO2* probe stained many neurons dark brown, the *PIEZO1* probe showed much fainter punctate staining. At the higher magnification (40× objective) some larger diameter neurons showed minimal or no staining by the *PIEZO1* probe but displayed light to heavy staining by the *PIEZO2* probe (red arrows in Figures 1C,D). In comparison, smaller neurons (i.e., see within red circle in Figure 1C) showed clear punctate staining by the *PIEZO1* probe while larger neurons in the same region (see red circle in Figure 1D) showed strong *PIEZO2* probe staining.

**FIGURE 2:**
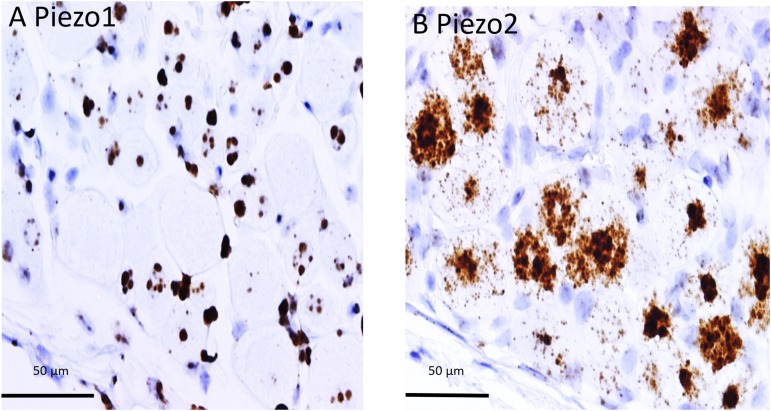
Comparison of *PIEZO1* and *PIEZO2* expression examined at high magnification (60× objective). Adjacent DRG slices stained with *PIEZO1* and *PIEZO2* probes indicating while most neurons express *PIEZO2* **(B)** several of the large neurons show zero or minimal *PIEZO1* expression **(A)**. Also the several small cells in **(B)** that surround neurons and appear universally stained blue with the hematoxylin counterstain are unstained by the *PIEZO2* probe **(B)** but at least some are heavily stained by the *PIEZO1* probe **(A)**.

The difference in *PIEZO1* and *PIEZO2* probe staining is shown even more clearly in Figure 2 for adjacent slices taken from another DRG and examined at still higher magnification (60× objective). Again, there appeared to be zero *PIEZO1* expression in some of the largest neurons but clear punctate staining in smaller neurons (Figure 2A). In comparison, *PIEZO 2* expression was evident in close to all of the largest neurons (i.e., ∼95%) and in most (i.e., >80%) of the smaller neurons (Figure 2B). The apparent ubiquity in *PIEZO2* expression meant that a significant proportion (>50%) of *PIEZO1*-expressing neurons also showed *PIEZO2* co-expression (see below).

### *PIEZO1* Expression as a Function of Cell Size

**FIGURE 3:**
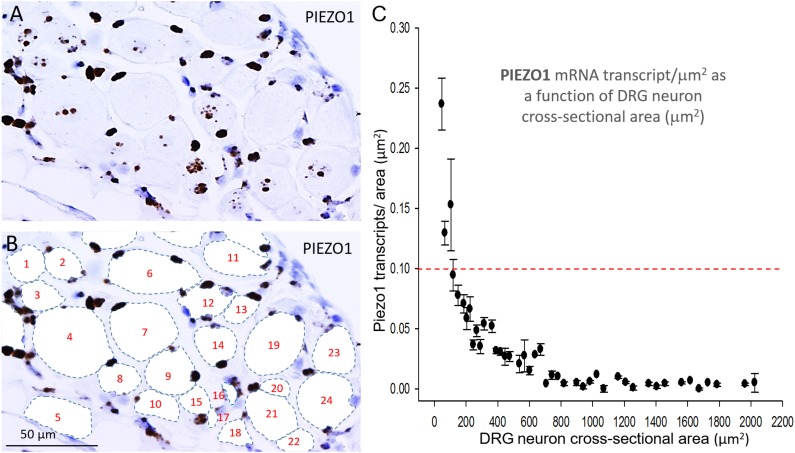
*PIEZO1* expression varies with DRG cell size. **(A)** DRG cells stained with the PIEZO1 RNA probe. Note the absence of *PIEZO1* expression in many of the largest neurons in contrast to relatively high expression in some of the smallest neurons. **(B)** The same microscopic field in which each neuron’s perimeter has been traced out to better define their cross-sectional area which has been numbered for identification. Following this procedure, individual neurons can be seen to be “ringed” by several smaller cells, consistent with satellite glial cells, that are heavily stained by the *PIEZO1* probe. **(C)** A plot showing the relationship between *PIEZO1* transcript densities normalized for neuronal cross-sectional area (transcripts/μm^2^) plotted as a function of DRG neuronal cross sectional area (μm^2^). The data represent transcripts counted in 167 neurons with each data points representing the mean ± SEM for 2–7 neurons. The red dashed line at 0.1 transcript/μm^2^ is drawn for comparison with the median expression of the house keeper gene PPIB (e.g., see Figure 4).

In order to measure the cross sectional area and *PIEZO1* expression of specific neurons, each neuron within a microscopic field had its membrane perimeter traced and its enclosed area numbered for identification (e.g., Figures 3A,B). Following this procedure, individual neurons could be clearly seen to be ringed by one or more darkly stained cells that were consistent with cell bodies of satellite glial cells (SGCs). For example, the neuron designated # 6 is ringed by at least 3 SGCs densely stained by the *PIEZO1* probe, whereas neuron #24 is associated with at least one darkly stained SGC and one unstained SGC that appears light blue from the hematoxylin counterstain. This heterogeneity in SGC staining by the *PIEZO1* probe may indicate a stochastic, all-or-none process regulates *PIEZO1* expression in SGCs. In comparison, the *PIEZO2* probe failed to stain SGCs (e.g., see all blue SGCs in Figure 2B). Figure 3 also confirms that specific large DRG neuron were either unstained by the *PIEZO1* probe (neurons 5, 7, 11, 19, and 23) or only showed relatively low density punctuate staining (neuron 4, 9 and 24). In contrast, several smaller neurons in the same field showed high density *PIEZO1* probe staining (neurons 1, 2, 3, 14, and 15). Figure 3C quantifies this dependence of *PIEZO1* expression on neuron size by plotting *PIEZO1* transcript density as function of neuronal cross-sectional area. The larger neurons ranging from ∼700 to over 2000 μm^2^ showed a very similar very low *PIEZO1* transcript density (0.006 ± 0.0005, *n* = 96). In contrast, smaller neurons from 500 to ∼100 μm^2^ showed a progressively increasing transcript density with the smallest (≤150 μm^2^) expressing a *PIEZO1* transcript density (0.19 ± 0.043, *n* = 23, *P* < 0.0001) that was more than 30-fold higher than measured in the larger neurons.

**FIGURE 4:**
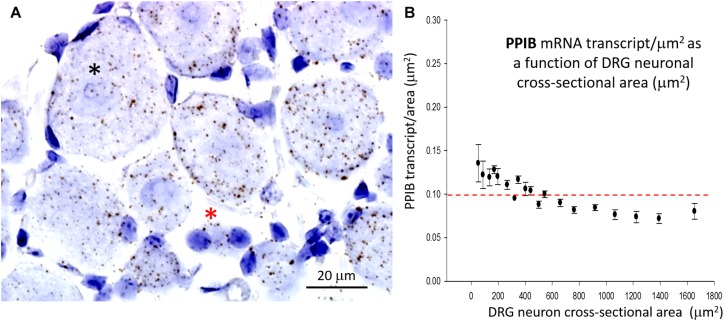
Expression of the housekeeper gene PPIB varies little with cell size. **(A)** DRG section showing qualitatively similar densities of PPIB transcripts in the largest (^*^) and smallest (^*^) neurons. PPIB transcripts at a similar density were also evident in the blue stained SGCs that surround neurons. **(B)** Plot of PPIB transcript density (transcripts/μm^2^) in neurons measured as a function of neuronal cross sectional area (μm^2^).

In marked contrast to the cell size-dependent PIEZO1 expression, the housekeeper gene peptidylprolyl isomerase B (*PPIB*) expression shows little cell size-dependence [e.g., compare designated large (^*^) and small (^*^) neurons in Figure 4A] with a less than 1.5-fold change in transcript density over the same neuronal size range (see Figure 4B). This difference between *PIEZO1 and PPIB* probe staining as a function of cell size is evident in the spreads of their data sets (c.f., Figures 3C, 4B) around the dashed red lines at 0.10 transcripts/μm^2^ that represents the ∼median *PPIB* transcript density. Finally, examination of Figure 4A also indicates that SGCs showed qualitatively similar *PPIB* transcript densities as their neighboring neurons.

### Comparing *PIEZO1, PIEZO2*, and *TRPV1* Expression and Co-expression

**FIGURE 5:**
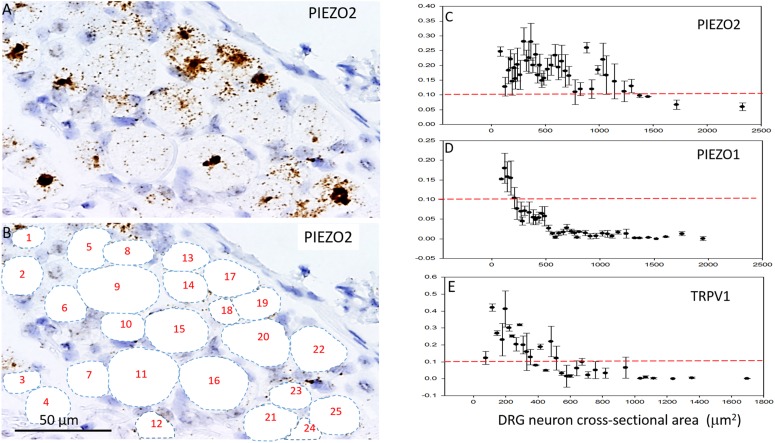
Comparison of *PIEZO1*, *PIEZO2*, and *TRPV1* expression in DRG. **(A,B)** A DRG slice stained with the *PIEZO2* probe with specific neurons identified and numbered for analysis. Note the variable density in *PIEZO2* probe staining with medium to smaller neurons showing stronger staining than larger neurons. **(C)** A plot showing the relationship between *PIEZO2* transcript densities normalized for neuronal cross-sectional area (transcripts/μm^2^) plotted as a function of DRG neuronal cross sectional area (μm^2^). The data represent transcripts counted in 200 neurons with each data points representing the mean ± SEM for 3–10 neurons. The red dashed line at 0.1 transcript/μm^2^ is drawn for comparison with the median expression of the housekeeper gene *PPIB.* **(D,E)** Similar plots as in **(B)** from the same DRG of *PIEZO1* and *TRPV1* probe staining. Note that the expression of *TRPV1* is similar to *PIEZO1* in that the larger neurons weakly express these genes. However, *PIEZO1* differs in that it is more selectively expressed in the smallest neurons compared with *TRPV1*.

Figures 5A–C shows analysis of *PIEZO2* expression as a function of neuron size, and indicates that *PIEZO2* is more widely and highly expressed in both small and large neurons than *PIEZO1* (c.f., Figures 5C,D). Indeed, the very high expression of *PIEZO2*, particularly in small and medium sized neurons, indicates significant *PIEZO2* co-expression (>50%) in those neurons that selectively express either *PIEZO1* (Figure 5C) or *TRPV1* (Figure 5E). TRPV1 is one of the channels strongly implicated in mediating heat-nociception (Caterina et al., 2000; Vandewauw et al., 2018) and is highly expressed in small and medium sized neurons but poorly expressed in large neurons (Figure 5E). This expression pattern appears similar to *PIEZO1*, although with overall greater expression in medium-sized neurons (c.f., Figures 5D,E). However, as described below the *PIEZO1*-expressing and *TRPV1*-expressing neurons represent essentially non-overlapping populations. It is evident that the expression density plots for the three genes (Figures 5C–E) show similar neuronal size distributions, indicating that the exclusive *PIEZO1* expression in the smaller SGCs did not bias the estimation of *PIEZO1* expression in neurons.

**FIGURE 6:**
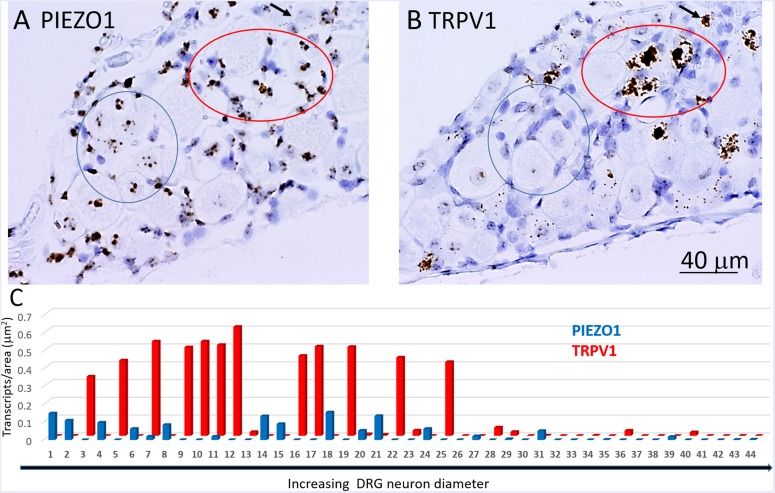
Co-expression of *PIEZO1* and *TRPV1* in a DRG. **(A)** DRG slices stained with the *PIEZO1* RNA probe. **(B)** The adjacent DRG slice stained with the *TRPV1* probe. The red oval designate a region in which at least three neurons are heavily stained with the *TRPV1* probe **(B)** whereas in the same region the *PIEZO1* probe stains only SGCs **(A)**. The blue ovals designates another region in which there is clear punctate staining by the *PIEZO1* probe **(A)** but no staining by the *TRPV1* probe **(B)**. The black arrow in the upper right quadrant in each panel designates on neuron that does show strong expression of *TRPV1* **(B)** and also punctate expression of *PIEZO1* **(A)**. Note the *TRPV1* probe, unlike the *PIEZO1* probe, does not stain SGCs. **(C)** A plot of 44 neurons identified in adjacent slices that were analyzed for *PIEZO1* and *TRPV1* co-expression. The 44 neurons are number according to increasing cross-sectional area with a range of 90-2070 μm^2^. Neuron #26 that showed no expression of *PIEZO1* or *TRPV1* had an area 565 μm^2^.

In order to determine co-expression of specific genes, individual neurons identified across adjacent DRG slices (i.e., stained with *PIEZO1*, *PIEZO2*, or *TRPV1* probes) were identified and analyzed. Figure 6 shows high magnified images of adjacent slices stained with the *PIEZO1* (6A) and *TRPV1* (6B) probes. The *TRPV1* probe stained very darkly several smaller and medium sized DRG neurons (e.g., see neurons enclosed within the red oval in Figure 6B). In contrast, neurons enclosed in the same region in Figure 6A showed little or no detectable staining by the *PIEZO1* probe even though many of the SGCs within the same region were strongly stained. Although most neurons that showed *TRPV1* staining did not show significant *PIEZO1* staining, at least one neuron in the same microscopic field (see the black arrow designated neuron in Figures 6A,B) darkly stained by the *TRPV1* probe also showed punctate staining by the *PIEZO1* probe. On the other hand, specific neurons that showed clear punctuate staining by the *PIEZO1* probe (e.g., within the blue circle in Figure 6A) showed little or no apparent staining by the *TRPV1* probe (see same region in Figure 6B). Figure 6C shows a plot describing the co-expression of *PIEZO1* and *TRPV1* in 44 neurons of progressively increasing cell size (range 150–1800 μm^2^ indicated by the extended black arrow). Again, the larger DRG neurons showed little or no expression of either *PIEZO1* or *TRPV1*, whereas, most of the smaller neurons expressed either *PIEZO1* or *TRPV1*. For example, some of the smaller neurons expressed only *PIEZO1* (neurons 1, 2, 4, 6, and 8), whereas of the 12 neurons that strongly expressed *TRPV1* (3, 5, 7, 9, 10, 11, 12, 16, 17, 19, 22, 25) only two neurons (7 and 11, 16%) showed detectable co-expression of *PIEZO1*.

**FIGURE 7:**
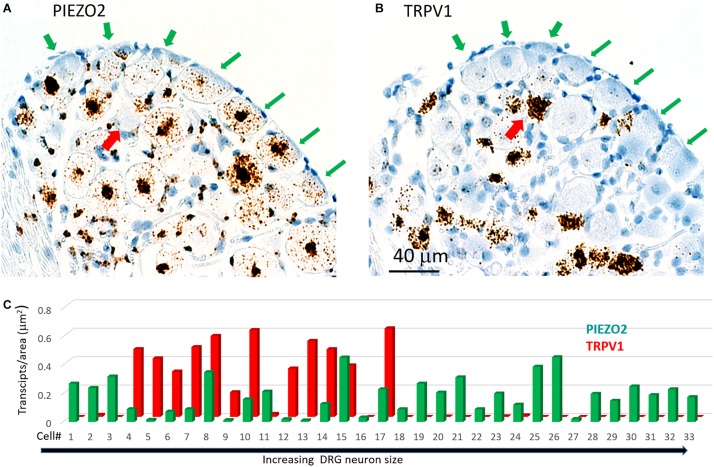
Co-expression of *PIEZO2* and *TRPV1* in DRG cells. **(A)** A DRG section stained by the *PIEZO2* probe. **(B)** The adjacent DRG section stained with the *TRPV1* probe. Note the selective expression of *PIEZO2* over *TRPV1*, particularly in the neurons indicated by the green arrows. Whereas almost all neurons in this section showed some staining by the *PIEZO2* probe (≥90%) less than 50% were stained by the *TRPV1* probe and these were the smaller neurons. The red arrow indicates at least one neuron heavily stained by the *TRPV1* probe that was not stained by the *PIEZO2* probe. **(C)** Analysis of co-expression of *PIEZO2* and *TRPV1* in 33 neurons identified in adjacent slices. The neurons were numbered in increasing cross-sectional area with a range of 100–1900 μm^2^. Neuron #18 had an area of 648 μm^2^ (Note these neurons are not the same as in Figure 6C).

Figure 7 shows another slice stained with *PIEZO2* and *TRPV1* probes. The microscopic fields indicate that many of the larger neurons that expressed *PIEZO2* showed either very little or no detectable *TRPV1* expression (c.f. green arrowed neurons in Figures 7A,B). In comparison, at least one identified neuron in the field that showed very high *TRPV1* expression (Figure 7B, red arrow) did not appear to express *PIEZO2* (Figure 7A). However, given the wide expression of *PIEZO2* in small and medium sized neurons (e.g., ∼85%) some neurons were seen that co-expressed *PIEZO2* and *TRPV1*. For example, of 33 neurons specifically analyzed for co-expression (Figure 7C), 12 of the smaller neurons showed *TRPV1* expression, and of these, eight neurons (i.e., 67%) showed at least some co-expression of *PIEZO2* (i.e., neurons 4, 6, 7, 8, 10, 14, 15, 17). Moreover, three neurons (i.e., 8, 15, and 17) showed high transcript density of both genes (i.e., ≥0.2 transcripts/μm^2^) consistent with multi-modal neurons. The numbered neurons analyzed in Figure 7C were in different DRG slices from the neurons analyzed in Figure 6C.

### Estimation of Specific Gene Expression Indices (ε) in Small and Large DRG Neurons

**FIGURE 8:**
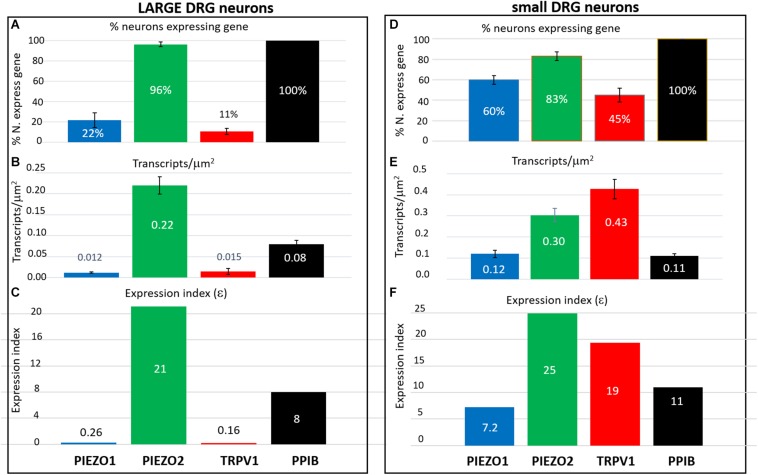
Expression index (ε) estimated for different genes in larger versus smaller DRG neurons. Left, Gene expression parameters estimated in analysis of 257 larger neurons (**≥**600 μm^2^). **(A**) Histograms showing the percentage of neurons expressing *PIEZO1, PIEZO2, TRPV1*, and *PPIB* genes. **(B)** Histograms showing the measured average gene transcript density (transcripts/μm^2^) in neurons that expressed the specific gene. **(C)** Histograms of the expression index calculated as the product of the % of neurons expressing the gene and their average transcript density (transcripts/μm^2^). Right, Expression parameters for 354 smaller neurons (≤500 μm^2^). **(D)** Histograms showing the percentage of neurons expressing *PIEZO1, PIEZO2, TRPV1*, and *PPIB* genes. **(E)** Histograms showing their average transcript density (transcripts/μm^2^) in neurons that expressed the specific gene. **(F)** Histogram of the expression index calculated as the product of the percentage of neurons expressing the gene and their average gene density transcripts/μm^2^.

In order to estimate the possible contribution of specific genes to DRG neuron function, an expression index (ε) was calculated as the product of the percentage of neurons expressing the gene (i.e., ≥1 transcript) multiplied by the average transcript density (transcripts/μm^2^). For convenience, we arbitrarily divided DRG neurons into large (≥600 μm^2^, Figures 8A–C) and small neurons (≤500 μm^2^, Figures 8D,E). As expected, 100% of both large and small neurons expressed the housekeeper gene *PPIB*, but with only moderate average expression levels (∼0.1 transcripts/μm^2^) to give an average ε value of ∼10 in both small and large neurons. In comparison, 96% of large neurons and 83% of small neurons expressed *PIEZO2*, with respective high expression indices of 21 and 25, due to the relatively high transcript densities (0.22 and 0.3 transcripts/μm^2^). Therefore, based on ε values alone, *PIEZO2* would be expected to play a significant functional role in both small and large DRG neurons, and is consistent with a recent study showing that complete *PIEZO2* knock-out, in addition to abolishing touch and proprioception (see also references in section “Introduction”) also partially impairs nociception (Murthy et al., 2018). On the other hand, the relatively large ε value for *PIEZO1* in small (7.2) versus large (0.26) neurons, indicates that *PIEZO1* may be capable of providing functional redundancy in small but not large neurons when *PIEZO 2* is genetically removed (or absent as in humans). Finally, in the case of TRPV1, the high ε value (19) in a mostly non-overlapping population of neurons distinct from the neurons expressing *PIEZO1*, is at least consistent with the idea that these two channels may participate in different forms of nociception. However, as already mentioned a proportion of *TRPV1*-expressing neurons (∼70 %) also expressed significant *PIEZO 2* (Figure 7) implying the existence of multimodal neurons capable of transducing both heat and mechanical stimuli. This demonstration of co-expression of *PIEZO2* and *TRPV1* also provides a cellular basis for the finding that capsaicin can strongly inhibit PIEZO2-mediated mechanosensitive currents in TRPV1-expressing neurons (Borbiro et al., 2015).

### Yoda1 Injection Causes a Prolonged Hyperalgesia in Mice

**FIGURE 9:**
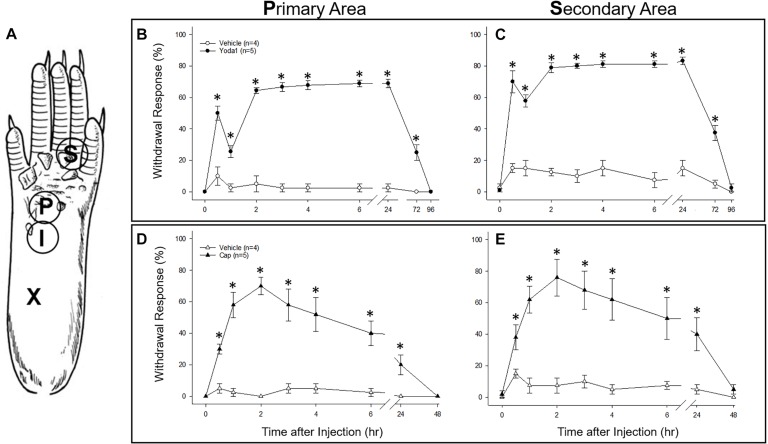
Comparison of nociceptive responses (mechanical hyperalgesia) induced in mice injected with Yoda1, capsaicin and vehicle alone. **(A)** A schematic showing sites of injection and behavioral testing in the mouse hind foot. For injection, a 30-gauge needle was inserted at the heel of the foot (X) and advanced to the injection site (I), where a 5 μl intradermal injection of Yoda1 (5 nanomole), capsaicin (15 nanomole) or vehicle alone was carried out. Foot withdrawal frequencies in response to von Frey filament stimuli were measured at the primary site (P) for hyperalgesia and at the secondary site (S) for hyperalgesia. **(B)** Primary and **(C)** secondary mechanical hyperalgesia measured in response to Yoda1 and vehicle alone. **(D)** Primary and **(E)** secondary mechanical hyperalgesia measured in response to capsaicin and vehicle alone. The data points represent mean ± SEM for 4–5 mice under each condition. The Mann–Whitney *U* test was used to compare Yoda1 or capsaicin against vehicle alone at each time point.

As an *in vivo* assay to determine the functional significance of the DRG neuronal specific expression of *PIEZO1* and *TRPV1*, we tested the responses in mice to injections of either capsaicin, a TRPV1 specific agonist (Caterina et al., 1997, 2000) or Yoda1, a PIEZO1 channel specific agonist and modulator (Syeda et al., 2015). In particular, Yoda1 is known to increase the mechanosensitivity of PIEZO1 channels, by both lowering their threshold and reducing their rapid inactivation in response to mechanical stimuli. Therefore, we tested mice for mechanical hyperalgesia, by measuring the nocifensive response to a mechanical stimulus, at a primary site (i.e., close ≤ 3 mm to the Yoda1 injection site) and at a secondary site that was relatively distant from the Yoda1 injection site (Figure 9A, and see section “Materials and Methods”). It has already been established in both mice and humans that capsaicin injections can induce mechanical hyperalgesia, as well as heat hyperalgesia, at the primary site, but only mechanical hyperalgesia at the secondary site (see Torebjörk et al., 1992; Schwartz et al., 2008, 2009). Figure 9 shows the results of 5 μl (5 nanomole) injections of Yoda1 (Figures 9B,C) and 5 μl (15 nanomole) capsaicin (Figures 9D,E) compared with 5 μl injections of vehicle alone. For Yoda1, significant mechanical hyperalgesia was evident 30 min after injection at both the primary (Figure 9B) and secondary sites (Figure 9C). However, from the early initial peak in hyperalgesia there was a significant decrease evident at 1 h, followed by return to even higher levels of hyperalgesia by 2 h. Most dramatically, this second phase of elevated hyperalgesia was still evident 72 h after the Yoda1 injection, and only fully recovered 96 h after the injection. All five mice tested with Yoda1 showed this response pattern of prolonged hyperalgesia with full recovery after only 96 h. The capsaicin response was different (Figures 9D,E) but similar to previously published capsaicin results (i.e., see Figure 2 in Schwartz et al., 2008). In particular, there was a progressive increase to a maximum hyperalgesia by 2 h at both the primary and secondary sites, but with capsaicin there was no early transient decline within the first hour. After the 2 h peak, the capsaicin-induced hyperalgesia progressively declined so that there was partial recovery at 24 h (unlike with Yoda1) but full recovery at both sites was only evident after 48 h. It is possible that the mechanical hyperalgesia caused by Yoda1 and capsaicin injections arises because of neurogenic inflammation (Richardson and Vasko, 2002). However, although Yoda1 did cause initial redness at the injection site, it was less pronounced than that caused by capsaicin. Furthermore, by 24 h after injection there was no sign of inflammation in either the Yoda1 and capsaicin injected groups. This observation alone may indicate that the mechanical hyperalgesia arises from central sensitization due to increased afferent nerve activity induced by Yoda1 in *PIEZO1-* expressing afferents (Mikhailov et al., 2019) or by capsaicin in TRPV1-expressing afferents (Banik and Brennan, 2009). However, a further possible complication is that keratinocytes, which are located in the epidermal layer of the skin, express PIEZO1 (Maksimovic et al., 2014) and TRPV1 (Ko et al., 1998). In this case, activation of keratinocytes could somehow contribute to the hyperalgesia by, for example, inducing an abnormal wound healing/inflammatory response (Pastar et al., 2008). However again, the absence of visible redness and inflammation 24 h after injection does not favor this as the major underlying mechanism for the prolonged hyperalgesia.

## Discussion

The key finding of this study is that *PIEZO1* expression shows a strong dependence on DRG neuron size, with a high expression index in small neurons (ε = 7.2) compared with large neurons (ε = 0.26). This contrasts with *PIEZO2*, which is highly expressed in most DRG neurons (∼90%) including both small (ε = 25) and large neurons (ε = 21). Consequently, a significant proportion of small neurons (∼60%) that express *PIEZO1* also co-express *PIEZO2*. This co-expression, may allow for chimeras of PIEZO1/PIEZO2 subunits to form a high threshold slow-inactivating channel that transduces painful stimuli, similar to the chimeric channels proposed to confer high-strain mechanosensitivity on articular cartilage (Lee et al., 2014). However, whereas knockout of either *PIEZO1* or *PIEZO2* abolishes chondrocyte mechanosensitivity (Lee et al., 2014) complete knockout of *PIEZO2* only partly impairs mechano-nociception (Murthy et al., 2018). Interestingly, a recent study of *PIEZO1* and *PIEZO2* expression in baroreceptor neurons of mouse visceral ganglia indicates an equal percentage of neurons (∼40%) expressed either *PIEZO1* or *PIEZO2*, but only a small percentage (∼15%) showed co-expression (Zeng et al., 2018). Significantly, double knockout of both *PIEZOs* was required to suppress the baroreflex-mediated heart rate changes (Zeng et al., 2018). Our results predict that a double knockout of *PIEZO1* and *PIEZO2* will also be required to block mechano-nociception.

**FIGURE 10:**
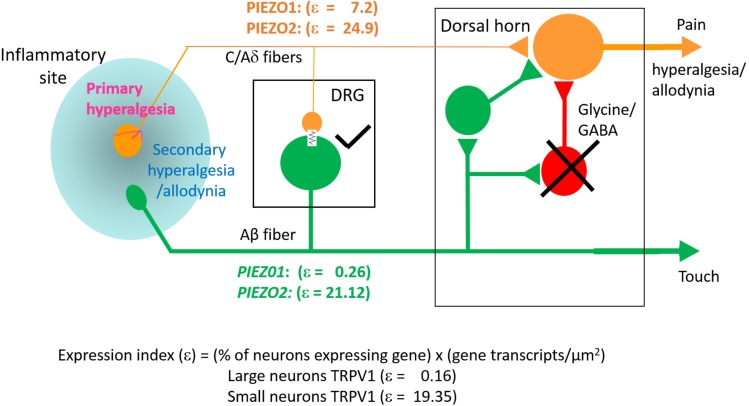
Schematic illustrating differential *PIEZO1* and *PIEZO2* expression in nociceptive and touch labeled lines and their predicted contributions to normal and abnormal pain states (allodynia and hyperalgesia). Touch is mediated by large cell body/thick axon myelinated DRG neurons (Aβ fibers) that express predominately *PIEZO2* with minimal expression of *PIEZO1*. Nociception is mediated by small body/thin axon unmyelinated (C) fibers and thinly myelinated (Aδ) fibers that express *PIEZO2* and relatively high levels of *PIEZO1*. As discussed in the text, hyperalgesia and allodynia are pathological pain states that may arise from crosstalk between the nociceptive and touch lines in addition to possible sensitization of peripheral nerve endings. Different mechanisms may promote this cross talk. One mechanism may occur within the DRG where inflammation, nerve damage and/or elevated neural activity induces gap junction connections (indicated by the electrical resistance symbol) between large and small diameter neurons. Another mechanism may occur within the dorsal horn in which the normal inhibitory synaptic input (indicated by the cross) that suppresses the influence of excitatory inputs on projecting neurons that transmit pain signals to the brain is reduced. In both cases, signals generated by *PIEZO2* activation in the “touch line” may also be transmitted to the “nociceptive line” (see text).

While *PIEZO2* knockout does not abolish mechano-nociception, there is substantial evidence for PIEZO2 involvement in transducing specific chronic pain states including hyperalgesia and allodynia (Dubin et al., 2012; Eijkelkamp et al., 2013; Singhmar et al., 2016; Yang et al., 2016; Murthy et al., 2018; Szczot et al., 2018). Whereas mechanical hyperalgesia, in the strict sense, involves an increased sensitivity to normally noxious mechanical stimuli, mechanical allodynia occurs when a normally non-noxious mechanical stimulus causes pain (Sandkühler, 2009). Two recent studies focused on mechanical allodynia – one involving *PIEZO2* knockout mice (Murthy et al., 2018), the other human patients deficient in *PIEZO2* expression (Szczot et al., 2018) – have shown that the absence of PIEZO2 completely eliminates the mechanical allodynia induced by capsaicin injection. Several mechanisms may underlie allodynia, including peripheral and central sensitization of nociceptive neurons (Sandkühler, 2009). In particular, allodynia may arise because of induced cross talk between touch and nociceptor labeled lines (see Figure 10) via induced gap junction communication between small and large DRG neurons (e.g., see Kim et al., 2016; Spray and Hanani, 2019). Another non-exclusive mechanism may arise via the removal of the inhibitory drive that normally suppresses excitatory synaptic inputs linking touch inputs to the pain projection neurons within the dorsal horn of the spinal cord (e.g., see Torsney and MacDermott, 2006; Arcourt et al., 2017). In terms of these mechanisms, one can see how genetic knockout of *PIEZO2* would eliminate allodynia while only partly impairing normal nociception because PIEZO1 may serve a redundant role (Figure 10). On the other hand, complete *PIEZO1* knockout should leave allodynia intact unless it occurs via sensitization of PIEZO1 channels in nociceptive nerve endings, while normal nociception may be only partly impaired, since in this case PIEZO2 serves redundant roles in both touch and mechano-nociception. Interestingly, a recent *PIEZO2* knockout study has reported that not only is there an impairment in touch but also an actual increase in normal mechanical nociception (Zhang et al., 2019). This paradoxical result is consistent with the idea that touch normally suppresses pain (Torsney and MacDermott, 2006; Arcourt et al., 2017). Moreover, following ectopic expression of *PIEZO1* in the *PIEZO2* knockout, not only was defective touch rescued, but mechanical pain was also suppressed, indicating that ectopic PIEZO1 can take over PIEZO2 function in large neurons (Zhang et al., 2019). These combined studies, considered from an evolutionary perspective, indicate how the *PIEZO* gene duplication that occurred in vertebrates – invertebrates like *Drosophila* express only one *PIEZO* and this forms the mechano-nociceptive channel (Coste et al., 2010; Kim et al., 2012) – may have given vertebrates an added selective advantage by introducing more redundancy and flexibility in transducing different forms of somatosensory stimulation.

**FIGURE 11:**
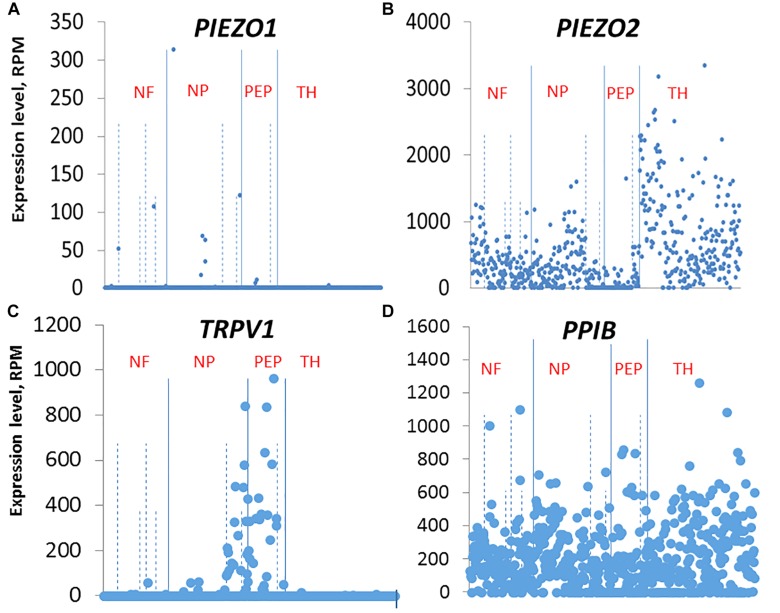
Single cell RNA-sequencing scatter plots for *PIEZO1* **(A)**, *PIEZO2* **(B)**, *TRPV1* **(C)**, and *PPIB* **(D)** taken from Usoskin et al. (2015) (see http://linnarssonlab.org/drg/). The vertical axis represents the normalized gene expression levels in reads per million (RPM) for individual cells. The RPM counts are grouped along the horizontal axis according to identified populations separated by the solid vertical lines in the order: NF, NP, PEP, TH populations (NF, Neurofilament; NP, Non-Peptidergic; PEP, Peptidergic; and TH, tyrosine hydroxylase). The dashed vertical lines separate major populations into further subtypes, for example NF1 to NF5 for NF major type.

Our ISH results indicate that the small DRG neurons that selectively express *PIEZO1* are part of a non-overlapping population mostly distinct from those neurons that strongly express *TRPV1*, one of the channels implicated in heat-nociception. This idea of distinct nociceptor populations for mechano- and heat-nociception in the DRG is consistent with several previous studies that used genetic and pharmacological approaches to selectively ablate TRPV1+ neurons and G-protein coupled receptor MRGPRD+ neurons, and demonstrate these neurons mediate heat- and mechano-nociception, respectively (Cavanaugh et al., 2009, see also Beaudry et al., 2017). Our results also agree qualitatively, if not quantitatively, with the single cell RNA-sequencing dissection of mouse DRG neurons^2^ (Usoskin et al., 2015). In this study, DRG neurons were classified into four distinct clusters: An NF cluster that expressed the neurofilament heavy chain associated with myelinated neurons. A PEP cluster that expressed substance P and calicitonin gene-related peptide associated with peptidergic nociceptors. An NP non-peptidergic cluster that was also *TRPV1*-negative, associated with mechano-nociceptors, and a TH cluster that expressed tyrosine hydroxylase (*Th*), and is associated with unmyelinated neurons (type C) involved in mediating pleasant or emotional touch. In terms of these four clusters, *PIEZO1* was expressed mainly, although at very low levels in the NP cluster (Figure 11 shows the relevant gene scatter plots taken from http://linnarssonlab.org/drg/ see also External resource Table 1). Specifically, seven of the 19 neurons that expressed *PIEZO1* (out of 622 neurons) were in the NP cluster (Figure 11A), and of those 19, 16 co-expressed *PIEZO2*. *PIEZO2* was widely expressed in all DRG neurons, but most predominately in the *Th* cluster (Figure 11B). As expected, *TRPV1* expressed almost exclusively in the PEP cluster involved in sensing noxious heat, although with some overlap into the NP cluster (Figure 11C). Moreover, of the 66 neurons that expressed *TRPV1*, 50 neurons co-expressed *PIEZO2.* In the case of non-neuronal cells, Usoskin et al. (2015) reported that only three out of ∼100 non-neuronal cells analyzed expressed *PIEZO1*. However, in two of these the *PIEZO1* transcript counts were several orders of magnitude higher than the counts in any neurons. At this time, we have no explanation for the quantitative differences in *PIEZO1* expression in mouse DRG seen between the ISH and scRNA-seq studies. However, our ISH analysis did appear to include more small DRG neurons (i.e., ≤10 μm in diameter e.g., see Figure 3B) than those analyzed in the scRNA-seq study (i.e., ≥15 μm in diameter).

A specific prediction from our ISH results is that Yoda1, a chemical identified as a highly selective agonist/modulator of PIEZO1 channels versus PIEZO2 channels (Syeda et al., 2015; Lacroix et al., 2018) should also produce some form of nociceptive response in mice. Indeed, the mechanical hyperalgesia we observed is consistent with a prolonged increase in mechanosensitivity and reduction in rapid channel inactivation of PIEZO1 channels that would tend to increase afferent nerve firing (Syeda et al., 2015; Lacroix et al., 2018). In direct support of this idea, a Finnish group (Mikhailov et al., 2019) has recently shown that Yoda1 induces rapid and large Ca^2+^ transients in isolated trigeminal *PIEZO1*-expressing neurons. Even more compelling, Yoda1 also induced, in a rat hemi-skull preparation, a pronounced and sustained firing of trigeminal mechanosensory nerve fibers innervating the meninges (Mikhailov et al., 2019). The last result may directly contribute to mechanical hyperalgesia we observe, since sustained firing of afferents alone can induce central sensitization and chronic pain states in both rodents and humans (Xie et al., 2005; Sandkühler, 2009; Pfau et al., 2011). Nevertheless, at this time off-target effects of Yoda1 cannot be excluded (Dela Paz and Frangos, 2018). A further added complication is that epidermal keratinocytes also express relatively high levels of PIEZO1 (Maksimovic et al., 2014). In this case, Yoda1 activation of keratocytes may induce an abnormal inflammatory response (Pastar et al., 2008; Shipton, 2013) that could underlie the resurgence in mechanical hyperalgesia (i.e., after ∼1 h) as well as the delayed recovery of hyperalgesia to baseline levels (i.e., >72 h, see Figure 8). However, again although Yoda1 did cause initial redness at the injection site, it was less pronounced than that caused by capsaicin. Furthermore, by 24 h after injection, there was no sign of inflammation in either the Yoda1 and capsaicin injected groups.

In conclusion, our DRG study and the recently published TG study (Mikhailov et al., 2019) directly implicates PIEZO1 in mechano-nociception. Obviously, further genetic manipulations of both *PIEZO1* and *PIEZO2* and will be required to confirm their individual and combined roles, as has been recently demonstrated for baroreception (Zeng et al., 2018). Interestingly, while in mice global knock-out of *PIEZO1* is embryonically lethal, in humans a PIEZO1 loss-of-function mutation has been reported to cause mainly a loss of lymphatic function (Lukacs et al., 2015) while a PIEZO1 gain-of-function mutation results in a red blood cell dehydration (e.g., see Ma et al., 2018). Although nociception changes were not reported, these disorders may not be associated with either impairment or enhancement of nociception because of the redundant role played by PIEZO2. Finally, from a biophysical perspective, a mechanism is required to explain how PIEZO channels, characterized by their very rapid inactivation (<10 ms), can mediate the slow inactivating currents that transduce mechano-nociceptive stimuli. Interestingly, in a variety of cells types that express PIEZO-like mechanically gated cation channels, the channel can be switched permanently from a transient to a sustained gating mode (i.e., TM →SM) by strong mechanical stimulation (Hamill and McBride, 1997; Maroto et al., 2012; Bae et al., 2013). On the other hand, a switch in PIEZO1 channel gating in the reverse direction (i.e., SM → TM) also occurs with differentiation of mouse embryonic stem cells (Del Marmol et al., 2018 see also Soria et al., 2013). Understanding the basis of these gating switches could provide a novel approach to manipulating how mechanotransducers including mechano-nociceptors respond to mechanical stimuli.

## Ethics Statement

This study was carried out in accordance with the recommendations of the Animal Care and Use Committee at the UTMB and are in accordance with the NIH Guide for the Care and Use of Laboratory Animals. All experimental protocols were approved by the Animal Care and Use Committee at the UTMB and are in accordance with the NIH Guide for the Care and Use of Laboratory Animals.

## Author Contributions

OH conceived the study and carried out the ISH experiments. JW surgically isolated the DRG and carried out the Yoda1/capsaicin injections and behavioral assays. J-HL analyzed the behavioral results. OH wrote the manuscript. All authors read and approved the final manuscript.

## Conflict of Interest Statement

The authors declare that the research was conducted in the absence of any commercial or financial relationships that could be construed as a potential conflict of interest.

## Footnotes

1 https://imagej.nih.gov/ij/

2 http://linnarssonlab.org/drg/
